# Zeocin-Induced Adaptive Response in *Saccharomyces cerevisiae*: The Contribution of Priming Dose and Experimental Design

**DOI:** 10.3390/molecules31091500

**Published:** 2026-04-30

**Authors:** Teodora Todorova, Stephka Chankova

**Affiliations:** Institute of Biodiversity and Ecosystem Research, Bulgarian Academy of Sciences, 2 Gagarin Str., 1113 Sofia, Bulgaria; tedi_todorova@yahoo.com

**Keywords:** Zeocin, *Saccharomyces cerevisiae*, low dose, adaptive response, experimental design, double-strand breaks, mutagenic, recombinogenic effects

## Abstract

We aimed to clarify how the priming dose and the experimental design could affect the development of an adaptive response (AR) induced by low-dose Zeocin (Zeo) in *Saccharomyces cerevisiae* strains with differing genetic constitution. Constant-field gel electrophoresis was used for measuring double-strand breaks (DSBs) induction and DNA rejoining; for microbiological experiments, Zimmermann’s test was used for measuring survival fraction and genetic events. Favorable experimental conditions for the induction of AR in both D7ts1 and 551 strains were determined: the priming dose inducing about 20% lethality or at least a 1.5-fold increased DSB level, 45 min inter-treatment time, and recovery time of 30–45 min. Both strains developed well-expressed AR, measured by increased cell survival, but differed in their ability to develop AR, measured by reduction in DSBs. This discrepancy could be due to different DSBs rejoining rather than different DNA susceptibility, and the partial contribution of DSB repair to cell survival in the split-dose experiments. The frequency of mutagenic and recombinogenic events and DSB levels were lower in split-dose treatment. The development of AR depends on several factors: the magnitude of the priming dose, DNA susceptibility, the duration of the ITT window, the duration of recovery time, as well as genetic constitution of strains.

## 1. Introduction

**Figure 1:**
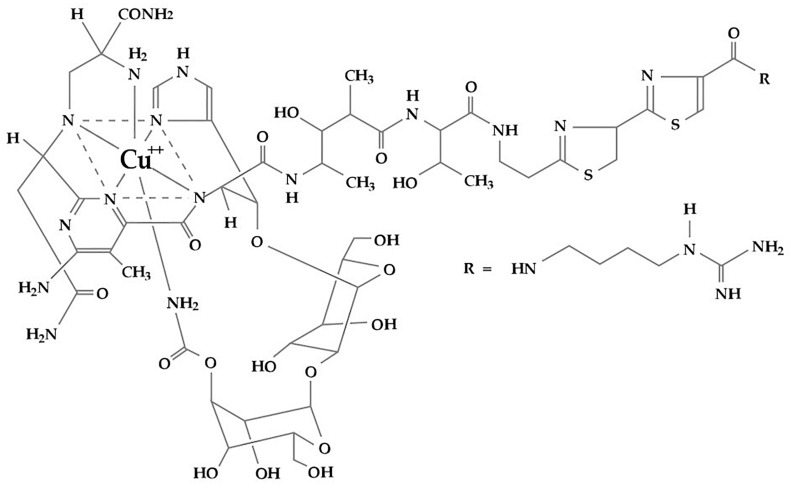
Chemical formula of Zeocin^TM^, adapted from Thermo Fisher Scientific Inc. (Waltham, MA, USA) User Guide.

Zeocin^TM^ (phleomycin D1) is a copper-chelated glucopeptide compound (Figure 1) belonging to the family of bleomycin antibiotics isolated from *Streptomyces verticillus* [1] with a similar mechanism of action as bleomycin. Entering the cell, the copper cation of Zeocin^TM^ (Zeo) is reduced to Cu^1+^ and removed by sulfhydryl compounds such as thiols in the cells [2]. Zeo was found to damage nucleosome-depleted and guanine-rich DNA regions [3,4]. They are characteristic regions with a high guanine–cytosine content. A large part of them is located in telomeres and in micro- and mini-satellite DNA [5].

In many papers, the large-scale damages induced by Zeo have been well described over the years. For example, Zeo’s pro-oxidative capacity [6,7,8,9,10,11] and its DNA-damaging potential, inducing DNA single- and double-strand breaks [4,6,9,12,13,14,15,16,17], have been revealed. Various other damages like point mutations, base losses—apurinic/apyrimidinic sites, large-scale chromosomal rearrangements, enhancement of the mutation rate, and alteration of the spectra of base substitutions at the whole-genome level (aneuploidy, loss of heterozygosity, as well as carcinogenic properties) were found [4,8,18,19,20]. Both up-regulation of specific DNA damage response (DDR) factors and down-regulation of cell cycle and histone genes were defined after Zeo application [11], completely blocking nuclear and cellular divisions [21].

In addition to the information mentioned above, our data revealed that Zeo, a well-known radiomimetic, can be used as an effective trigger to induce the phenomenon named “adaptive response” (AR) in *Chlamydomonas reinhardtii* strains [14,15]. Later, this information was confirmed by other authors [6]. The adaptive response is an evolutionarily developed mechanism that protects cells or organisms from radiation and harmful environmental conditions. A minimal stress is required (a low dose of radiation or chemical agent, called a “primary” dose, causing low levels of damage), which could activate various defense systems for subsequent protection against a similar or different high-level stress—a “test” dose [14,22,23,24,25,26,27].

Both extensive and in-depth information on the main mechanisms probably involved in the formation of AR, the different endpoints used for its assessment, the many organisms developing AR, as well as its importance to humans and the environment can be found in [23,27,28,29,30,31,32,33].

Despite the fact that the AR has been in the focus of scientists for several decades, the contribution of many items like genotype differences [15,26,29,34,35,36,37,38,39,40], effect of priming dose and experimental design [40,41,42,43,44,45,46], as well as DNA repair capacity [14,15,25,38,40,47,48,49,50] needs further clarification.

Based on the abovementioned findings and our own data on *Chlamydomonas reinhardtii* strains, here, we hypothesized that both the priming dose as well as experimental design contribute to a great extent to the formation of an AR. To clarify this, *Saccharomyces cerevisiae* was chosen as a model system.

Yeasts have a long-term history as models for studying the formation of AR to different stressors—natural (ionizing radiation and solar ultraviolet light) or ones released in the environment (anthropogenic pollutants). Some significant results concerning the role of protein synthesis [51], including specific heat shock protein HSP104 [52], homologous recombination [53], and DNA repair capacity [54] have been reported. Boreham et al. [55] suggested that the signal for the induction of radioresistant genes is more likely to arise from local distortions in DNA topology than from DNA damage. Later, another study pointed out that priming *Saccharomyces cerevisiae* cells with low-dose gamma-ray radiation alters cell cycle progression and time-dependent changes in the expression of genes *SSA1*, *SSA2*, *SSA4*, *KAR2*, *MRE11*, *RAD50* and *XRS2*, indicating the role of the unfolded protein response (UPR) pathway together with the Mre11-Rad50-Xrs2 (MRX) complex [56,57]. The effect of the priming dose has been reported in studies with gamma-ray irradiation [58].

Furthermore, as noted above, Zeo preferentially damages guanine-rich regions, such as G4 structures. Their genomic location is evolutionarily conserved between humans and yeast [5]. Nevertheless, yeast experiments provide significant insights into cellular responses, and the results can be readily extrapolated to higher eukaryotes, including humans, given the similarities in pathways. The major benefit is the model’s simplicity, which overcomes the ethical constraints [59].

In this work, we have focused our attention on two main questions, aiming to shed more light on how the priming dose, as well as the experimental design, could affect the development of an AR induced by low-dose Zeo in *S. cerevisiae* strains that differ in their genetic constitution.

## 2. Results

### 2.1. The Role of Genetic Constitution After a Single-Dose Treatment with Zeo

**Figure 2:**
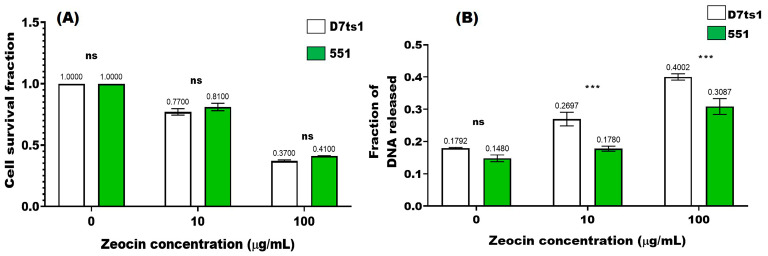
Stress response of *Saccharomyces cerevisiae* strains D7ts1 (white) and 551 (green) to the single Zeo treatment with two concentrations of 10 µg/mL and 100 µg/mL. (**A**) Cell survival response; (**B**) levels of primary-induced DSBs. For the cell survival study, the Petri dishes were plated immediately after the treatment, and the cell survival was counted on day 3. Data are mean values from nine independent experiments. Where standard errors are not visible, they are equal to or less than the symbols on the plots. The statistical significance of the responses between the two strains was assessed using a two-way ANOVA with Tukey’s post hoc test (ns: *p* > 0.05; ***: *p* < 0.001).

As a first step in our investigation, we have compared the responses of both strains, D7ts1 and 551, to a single Zeo treatment using two endpoints: cell survival and DSB induction (Figure 2A,B). All results were statistically significant compared to the control samples (untreated cells; *p* < 0.0001). Analyzing the data presented in Figure 2, two main findings should be pointed out: a concentration-dependent decrease in cell survival for both strains (Figure 2A), and approximately similar levels of cell survival between both strains after the treatment with 10 µg/mL and 100 µg/mL Zeo.

With respect to the other endpoint—DNA susceptibility, measured as DSB levels induced after Zeo treatment—the picture was quite different. Around 1.5-fold higher DSB levels (*p* < 0.001) were calculated after the treatment with 10 µg/mL in strain D7ts1 (Figure 2B), while no statistically significant increase was obtained for the haploid one—551 (*p* > 0.05). Single-dose treatment with 100 µg/mL was found to enhance DSB levels in a similar way for both strains—2.23-fold higher compared to the control levels for D7ts1 (*p* < 0.0001) and a 2-fold increase for 551 (*p* < 0.0001). This minor difference between 2.23-fold and 2-fold was statistically significant (*p* < 0.001). Correlation analysis revealed a strong negative correlation between cell survival and DSB levels in strain D7ts1 (r = −0.999, *p* < 0.05). On the other side, such a negative correlation was not statistically significant for strain 551 (r = −0.990, *p* > 0.05).

### 2.2. The Contribution of Inter-Treatment Time (ITT) to the Magnitude of AR Measured as Survival Fraction (SF) and DSB Levels When Split Treatment Was Performed

#### 2.2.1. Cell Survival Fraction (SF)

**Figure 3:**
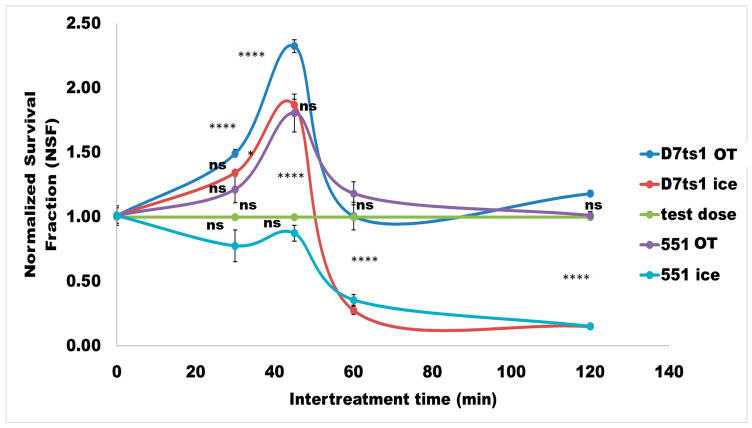
The effect of inter-treatment time (30, 45, 60, and 120 min) and incubation conditions (at optimal temperature (OT) and on ice) on the magnitude of Zeo-induced AR measured as cell survival fraction of both strains—D7ts1 and 551. Priming dose (10 µg/mL); test dose (100 µg/mL) of Zeo; data are presented as a normalized cell survival fraction (NSF). Point 1.00 on the ordinate scale indicates cell survival fraction after a single treatment with the test dose. Samples are as follows: D7ts1 incubated at optimal temperature (dark blue); D7ts1 on ice (red); 551 at optimal temperature (lilac); and 551 on ice (pale blue). The cell survival was counted on day 3. Data are mean values from nine independent experiments. Where standard errors are not visible, they are equal to or less than the symbols on the plots. The statistical significance among the curves for the two strains was assessed using a two-way ANOVA with Tukey’s post hoc test (ns, *p* > 0.05; *, *p* < 0.05; ****, *p* < 0.0001).

Different ITTs, in the range of 30 to 120 min, were used in split experiments to evaluate the optimal ITT for the induction of an AR. The presented results were normalized, as described in Section 4. Point 1.00 on the ordinate scale indicates the cell survival fraction after a single Zeo treatment with the test dose (100 µg/mL). In Figure 3, the differences in SF between the two strains were evident. Analyzing both curves, it is clear that a 45 min ITT was favorable for cells’ SF under optimal temperature (30 °C), with a 2.3-fold increase for D7ts1 (*p* < 0.0001) and a 1.8-fold increase for 551 (*p* < 0.0001). This difference was statistically significant (*p* < 0.0001).

A similar experimental scheme was performed on ice to measure SFs at experimental conditions probably preventing DSB repair (Figure 3). A huge difference was identified comparing the curves of both strains (see the curve in red for D7ts1 and pale blue for 551). Keeping D7ts1 on ice for 45 min did not abrogate to a full extent the cells’ capability to survive when split treatment was given. It could be assumed that some repair processes in D7ts1 remained active despite the incubation on ice. Contrarily, the SF of strain 551 was dramatically decreased at the same experimental conditions on ice.

#### 2.2.2. The Kinetics of DSB Levels in Split Experiments, Depending on the ITT

**Figure 4:**
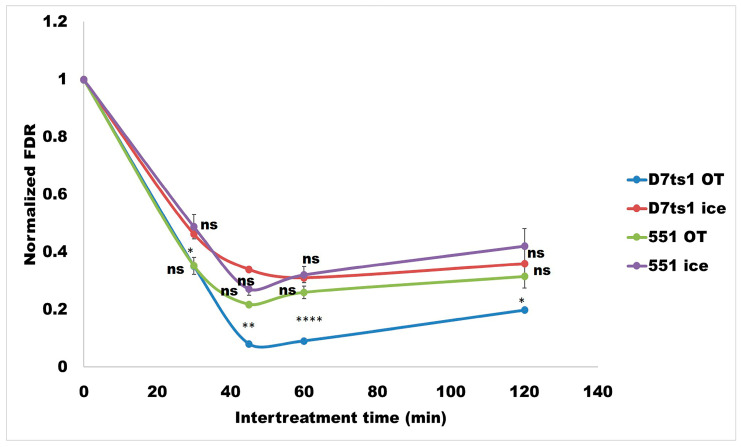
The kinetics of DSBs measured in both strains—D7ts1 and 551—depending on the ITT time—30, 45, 60, and 120 min—at optimal temperature and on ice after split treatment with priming (10 µg/mL) and a test dose (100 µg/mL) of Zeo. DSB levels are presented as a normalized Fraction of Damage Released (FDR). Point 1.00 on the ordinate scale indicates FDR after a single treatment with the test dose. Samples are as follows: D7ts1 incubated at optimal temperature (dark blue); D7ts1 on ice (red); 551 at optimal temperature (green); and 551 on ice (lilac). Where standard errors are not visible, they are equal to or less than the symbols on the plots. The statistical significance of the responses between both strains was assessed using a two-way ANOVA with Tukey’s *post hoc* test (ns *p* > 0.05; * *p* < 0.05; ** *p* < 0.01; **** *p* < 0.0001).

The data shown in Figure 4 illustrate the differences in DSB kinetics between both strains, depending on the ITT and the incubation conditions (on ice or at the optimal temperature—OT). Looking at Figure 4, it is evident that the remaining DSBs strongly depend on the ITT, but in all split-treated samples, they were lower than those measured after treatment with a single test dose of 100 µg/mL (*p* < 0.0001). The 45 min ITT was shown to favor the reduction in DSB levels.

A well-defined difference between the slopes of the two curves was obtained for strain D7ts1, when cell samples were kept at optimal temperature and on ice (blue curve—sample kept at optimal temperature; red curve—sample kept on ice). The most pronounced effect of DSB reduction was measured when samples were incubated at the optimal temperature for 45 min. Some DSB reduction was also calculated when cells were incubated on ice, probably due to the fact that DSB repair was not completely inhibited on ice (Figure 4).

The levels of DSBs in split experiments were also lower for strain 551, compared with those after a single test dose treatment. Here, it should be pointed out that no statistically significant differences between the green curve—samples at OT—and the lilac curve—samples on ice—were calculated.

### 2.3. The Contribution of the Recovery Time (RT)

To assess the role of the recovery time given after the test dose on the kinetics of double-strand break repair, new experiments were performed as follows: initial dose of 10 µg/mL Zeo >>> 45 min ITT >>> test dose of 100 µg/mL Zeo >>> RT—30, 45, and 60 min.

**Figure 5:**
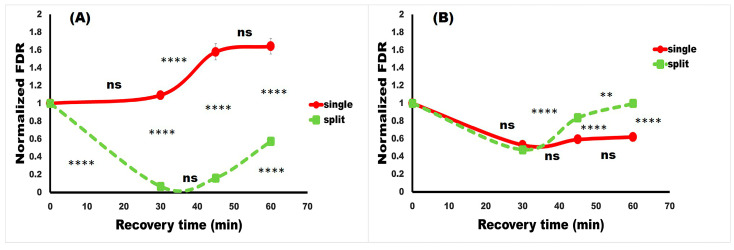
The effect of the recovery time (RT) for the formation of an AR in the diploid strain D7ts1 (**A**) and 551 (**B**), assessed by comparison of the single test dose (100 µg/mL Zeo) treatment (red) and split treatment (green) with Zeo (priming dose—10 µg/mL, 45 min ITT, test dose—100 µg/mL); recovery time (30, 45 and 60 min). DSB levels are presented as a normalized Fraction of Damage Released (FDR). Point 1.00 on the ordinate scale indicates FDR after a single treatment with the test dose Zeo. Where standard errors are not visible, they are equal to or less than the symbols on the plots. Data are mean values ± standard error of mean from at least eight experiments with independently grown cultures. The statistical significance of the responses between treatments for both strains was assessed using a two-way ANOVA with Tukey’s post hoc test (ns *p* > 0.05; ** *p* < 0.01; **** *p* < 0.0001).

The kinetics of DSB repair for both strains, depending on the RT given after the test dose, are presented in Figure 5. The differences between the red curve (single Zeo treatment with a test dose) and the green curve (split-dose experiments) are very pronounced in strain D7ts1 (Figure 5A). Both recovery times (30 min and 45 min) applied after the test dose Zeo treatment resulted in reduced DSB levels, probably due to accelerated DSB rejoining in D7ts1 experiments. It should be noted that no statistically significant difference was obtained between the two RTs. A slight increase in DSB was calculated 60 min after the test dose. It can be said that the 45 min is the most appropriate RT for this strain. These results indicate a well-developed AR, measured as reduced DSB levels (FDR) in D7ts1 split experiments. Supporting such a finding, data presented in Table 1 illustrate the role of RT in the magnitude of AR of D7ts1, measured as DSB rejoining.

The same experiments with the strain 551 revealed another trend (Figure 5B). No effect of the RT was obtained for this strain, suggesting that in 551, no additional acceleration of DSB rejoining was activated during the recovery time. Surprisingly, the levels of DSBs were statistically significantly higher in split-dose experiments (green curve) compared with single-dose Zeo experiments (red curve). It is very complicated to explain this result. Speculating, it could be proposed that different responses of the two strains could be attributed to different genetic constitution (D7ts1—diploid and 551—haploid) and to different types of DSB repair involved—homologous for D7ts1 and non-homologous for 551.

### 2.4. Mutagenic and Recombinogenic Potential of Zeo After Single-Dose Treatment

The contribution of the priming dose and the experimental design to the formation of an AR was further evaluated. The results presented in Table 2 provide information on the mutagenic and recombinogenic potential of Zeo after single and split treatments.

The dose-dependent recombinogenic/mutagenic potential of Zeo was revealed by analyzing the frequencies of these genetic events in cells treated with 10 and 100 μg/mL of Zeo (Table 2). The frequency of mitotic gene conversion at the *trp5* locus increased 3-fold after a single treatment with 10 μg/mL and 30-fold after a single treatment with 100 μg/mL, compared with that in control, untreated cells. Similar effects were observed for the other two genetic events induced after the same treatments. The values for revertants were 2.5 and 18-fold higher, and for total aberrants, from 2.5 to 36-fold higher. These results indicated the pronounced mutagenic and recombinogenic effects of Zeo, even at low doses.

### 2.5. The Mitotic Gene Conversion, Reverse Mutations, and Mitotic Crossing-Over After Split-Dose Treatment of Strain D7ts1 with Zeo

To assess the contribution of the experimental design, the split-dose experiment was evaluated with respect to these genetic events.

When a 45 min ITT at an optimal temperature for cell growth was given, quite different results were obtained from those after a single treatment with both doses (Table 2). Analyzing the same mutagenic and recombinogenic events, a significant decrease in the frequency of convertants (around 2-fold lower, *p* < 0.0001), revertants (around 3-fold lower, *p* < 0.0001), and around 7-fold lower for the total aberrant (*p* < 0.0001) was calculated.

The next experimental scheme involved keeping the cells on ice during the ITT to prevent the activation of some defense mechanisms (Table 2). Interestingly, the measured levels were significantly lower than those observed with a single treatment with the test dose. Despite that, when comparing with the experiments performed at an optimal temperature, a statistically significant increase in the frequencies of convertants (1.7-fold), revertants (1.8-fold), and total aberrants (2-fold) was observed (*p* < 0.0001). These results again provide evidence that the repair mechanisms were not completely blocked during the 45 min ITT.

### 2.6. Correlation Among Cell Survival, DSBs Remaining, and Genetic Events in Split-Dose Treatment Experiments

It was found that the frequency of the three genetic events—total aberrants, reverse mutations, and mitotic gene conversion—positively correlated with DSB levels when the experiment was carried out with 45 min ITT and optimal temperature (Table 3). Additionally, the three genetic events were also correlated. A strong negative correlation was observed only between the decrease in DSB and the increase in cell survival (*p* < 0.05).

On the other hand, when ITT was performed on ice to prevent DNA repair, again, a strong inverse correlation between cell survival and DSB levels was observed (Table 4). On the contrary, not all the genetic events were correlated with the DSBs. The only statistically significant correlation was calculated for the reverse mutations (*p* < 0.05). Based on this, it could be suggested that the defense mechanisms when incubation is on ice differ from those at optimal temperature.

## 3. Discussion

The present work aimed to address how the low priming dose and the experimental design contribute to the formation of Zeo-induced AR in *Saccharomyces cerevisiae* strains differing in their genetic constitution. Here, we discuss our results in the light of present understanding regarding several items: (1) what exactly a low dose that can trigger an AR means; (2) the ITT window required to activate a cell’s defense mechanism, including the repair capacity of double-strand breaks (DSBs); and (3) the most favorable experimental conditions for the development of an AR measured by different endpoints—SF, DSB repair capacity after split experiments, and mutagenic and recombinogenic events scored.

The pivotal role of the low priming dose in the formation of AR has been well documented [14,15,22,23,24,25,26,27,34,41,42,43]. The concept for the meaning and importance of low priming dose was well discussed by Yonezawa [42,60]. A large amount of very different information was gathered over the years. For example, our previous experiments with different strains of *Chlamydomonas reinhardtii* revealed that increasing the initial dose to a certain threshold [25,26,40] correlates with the magnitude of the adaptive response and genotypic resistance, and even radio- and chemoresistant strains can develop an adaptive response. For the first time in *Chlamydomonas reinhardtii* strains, the priming dose that can trigger an AR was calculated as the dose that induces approximately a 1.5-fold increase in DSB levels relative to control levels [14,15,27].

Other experiments with the *S. cerevisiae* strain D7 revealed that the most effective priming dose of gamma-ray irradiation was up to LD_10_ [56]. Bala and Goel [61] reported that the UV-C pre-irradiation with a dose that causes ≤ 10% cell kill is the most effective at inducing resistance to lethal UV-C doses (LD_50_). Another study using *S. cerevisiae* cells found that the UV-C priming dose required to activate defense processes related to AR formation should induce mild stress [62]. In our experiments, it was shown that priming cells with a low concentration of Zeo, which induces about 20% lethality [16,17], is a sufficient approach to induce an AR, as measured by increased SF.

Subsequent experiments revealed a completely different picture when the induced AR was evaluated by double-strand break rejoining. It became clear that the diploid strain D7ts1 can develop a well-pronounced adaptive response, while the haploid strain 551 did not. Both strains were found to differ in their capability to develop an AR measured as increased DSB rejoining. The main difference between them was that the concentration of 10 µg/mL Zeo induced 1.5-fold higher levels of DSB in D7ts1, whereas no statistically significant increase in DSB levels was observed in strain 551.

Speculating, it could be proposed that such a difference could be addressed to different recombination repair pathways regulated by ploidy and the MAT loci [63,64,65]. In the haploid strains, NHEJ is more effective due to the lack of a homologous repair template in G1, while HR is preferable in diploids, due to the homologous repair template [66].

Several decades ago, it was predicted that small levels of DNA damage could initiate the development of an AR [14,25,27,67,68,69]. The results presented here with *Saccharomyces cerevisiae* strains are in good agreement with our previous finding in *Chlamydomonas reinhardtii* strains that 1.5-fold increased DSB levels in primed cells would be a triggering event for DSBs’ accelerated rejoining and contribute to the formation of an AR [14,15,27].

Another important factor in the formation of the AR was the window time between the two doses. Different studies on bacteria, unicellular eukaryotes, or cell lines have defined the time window [32] between both doses in the range of 10 min to several h [6,14,15,18,49,56,61,68,70,71,72,73,74,75], and even days and months for multicellular organisms [76,77]. Studies on *Saccharomyces cerevisiae* revealed variations in the ITT, ranging from 1 h when hydrogen peroxide was used as a low dose [72] to more than 4 h when gamma rays or UV-C were applied [56,61]. Given that the doubling time of *S. cerevisiae* is typically 90 min [78], when the ITT exceeds 90 min, the effects observed should already be considered responses of the next generation(s). That is why, in our experiments, the 45 min ITT provides significant information on the effect of Zeo within the same cell population. Our data illustrated the significant role of the ITT. The 45 min ITT given after a low-dose Zeo treatment was defined as the most favorable for both strains to survive. Based on cell SF, both strains are proficient at developing an AR, measured as an enhanced survival fraction.

As mentioned in the Introduction, different endpoints could be used to evaluate cells/organisms’ capacity to develop an AR under certain experimental/environmental conditions. The reduction in DSB levels in split treatment experiments compared to those measured after single test dose treatment was applied by us as a second endpoint. The experimental results presented in this article confirmed without doubt our previous finding in *Chlamydomonas reinhardtii* strains that both ITT and RT play a very significant role in inducing AR, and that an acceleration of DSB rejoining was identified in split experiments [14,15,25,26,27,40,49]. The most pronounced effect was calculated for strain D7ts1 compared to strain 551. Some DSB reduction was also calculated when cells were incubated on ice, probably due to the fact that DSB repair was not completely inhibited on ice.

The suitability of the experimental design applied by us was further confirmed by evaluating the other genetic events—mitotic gene conversion, reverse mutations, and total aberrants. When the proposed experimental design with 45 min ITT was applied, a pronounced reduction, approximately 6-fold, was observed in the percentage of total aberrants. The reverse mutations and mitotic gene conversion were reduced by approximately 2-fold compared with the levels measured after treatment with the test dose of Zeo.

It is known that mitotic gene conversion is associated with homologous recombination. The 2-fold decrease in the frequency of convertants could be explained by a slight increase in homologous recombination. The correlation between mitotic gene conversion and DSBs is expected, as DSBs induce it. This reduction in levels could serve as confirmation of the increase in DSB rejoining capacity. It is well-known that several repair pathways exist when DSBs are present. By now, the role of homologous recombination in the formation of an adaptive response has been highlighted [53,79,80]. It is known that homologous recombination is primarily active during the G_2_ phase. Interestingly, Dwivedi et al. [56] reported an increase in the G_1_ population immediately after priming dose irradiation. The increase in the S-phase population was observed after 3 h. In our studies, the most pronounced effect was observed with a 45 min ITT. As shown previously, although up-regulation of the *RAD50*, *RAD51*, and *RAD54* genes was observed after low-dose gamma irradiation, a greater effect on AR formation was attributed to the unfolded protein response pathway, together with the MRX complex [56]. Rad54p and Rad50p are involved in homologous recombination and the repair of double-strand DNA breaks [81]. Under conditions that prevent radio-induced resistance, *RAD50* was found to be down-regulated [61]. In our experiments, when cells were incubated on ice, the repair processes were not completely blocked, suggesting the involvement of several mechanisms.

Thus, trying to understand some of the mechanisms, we analyzed the other endpoints. The frequency of the reverse mutations correlated with that of total aberrants and with DSB levels. The *ilv1-92* allele is suppressible, and genetic evidence indicates that Ilv^+^ revertants arise from both base-pair substitutions and frameshift mutations [82]. The two-fold increase in revertants after priming the cells with a low dose is a marker of increased base-substitution levels. It is known that they lead to inhibition of the DNA replication and activation of the S-phase progression checkpoint [56]. Zhang et al. [83] reported that Zeo treatment leads to the collapse of the replication fork.

Among all the studied endpoints, the most influenced one was the total aberrants. The genetic events linked to the *ade2* locus, classified as total aberrants, include not only mitotic crossing-over between the centromere and the *ade2* locus, which is the predominant event, but also a small fraction that may be attributable to gene conversion at the *ade2* locus, point mutation, deletion, chromosome loss, etc. [84]. Because these events are related to various repair systems, it is reasonable to suggest that homologous recombination is not the sole factor in AR formation, and other repair systems, such as mismatch repair, base excision repair, nucleotide excision repair, and others, may also be involved.

## 4. Materials and Methods

### 4.1. Chemicals

Zeocin was purchased from Invivogen (San Diego, CA, USA, Cat.code: ant-zn-1). Nutritional components (Gibco™) for yeast media preparation were obtained from Thermo Fisher Scientific (Waltham, MA, USA). Chemicals and reagents were of analytical grade.

### 4.2. Strains

Two *Saccharomyces cerevisiae* strains were used—D7ts1 (*MATa/α*; *ade2-119/ade2-40*; *trp5-27/trp5-12*; *ilv1-92/ilv1-92*; *ts1/ts1*) [85] and 551 rho+ (*MAT*α, *ura3*, *his3∆200*:*TymHis3AI*, *sec53*, *rho^+^*) [86].

These strains were chosen based on several reasons:

- Increased cell wall permeability compared to the commonly used laboratory strains *S. cerevisiae*. One of the main obstacles in experiments with yeast is its thick cell wall, which is impermeable to mutagens and carcinogens [87]. The strains used in our work carry a temperature-sensitive mutation, ts1, that non-specifically increases the cellular permeability of *S. cerevisiae* to various substances [88], including mutagens [85,86]. It is known that this mutation represents a transition from cytosine to thymine, changing proline at the 5′ end of the *TS1* gene (identified as the *SEC53* gene) to leucine [87]. The main role of this gene is to encode phosphomannomutase, which is required for an early step in the pathway of O- and N-linked mannosylation [89]. The impairment of protein glycosylation in *ts1* results in increased permeability of the *S. cerevisiae* cell wall.
- Both strains are genetically constructed to detect different genetic events. Strain 551 carries a Ty1 retrotransposon with an inserted HIS3AI construct (a HIS3 gene with an artificial intron), which provides an advantage for detecting retrotransposition events in the genome [86,87]. Strain D7ts1 is specifically constructed to detect mitotic gene conversion in the *trp5* locus, reverse mutations in the *ilv1* locus, and mitotic crossing-over between the centromere and the *ade2* locus [82].
- Our previous data reported the effects of various Zeo concentrations on both strains. Results regarding cell survival of strain 551 were gained based on a concentration range of 2–300 μg/mL, while DSBs were measured after the treatment with 10, 50, 100, 200, and 300 μg/mL [16]; for strain D7ts1, cell survival was evaluated for a concentration range of 2–300 μg/mL and DSBs—10, 50, 100, 200, 300, and 400 μg/mL [17]. No differences in Zeo resistance of both strains were obtained using cell survival capacity as an endpoint—around 80% survival after the single treatment of 10 µg/mL—and the LD_50_ was similar—around 68 µg/mL [16,17].

### 4.3. Cultivation Conditions

Cell suspensions were cultivated under standard conditions (30 °C, 200 rpm) in rich liquid YEPD media (1% yeast extract, 2% peptone, and 2% dextrose) for at least 18 h. Our previous studies reported that *S. cerevisiae* strains have differential sensitivity to Zeocin depending on the growth phase [9,17]. The present experiments were performed with cell cultures at the end of the exponential phase and at the beginning of the stationary phase [16,17], as the most susceptible to Zeo treatment. All experiments were repeated nine times with independently grown cell cultures.

Cell density was determined optically at 600 nm. Depending on the methodology, cells were diluted to 1 × 10^7^ cells/mL (for microbiological experiments) or 1 × 10^6^ cells/mL (for molecular experiments).

### 4.4. Experimental Designs Applied

Step 1: Single-dose treatment to assess the DNA susceptibility of both strains:

To confirm our previous findings, two doses were tested: a low dose of 10 μg/mL, used in subsequent experiments as a priming dose, and a high dose of 100 μg/mL, used in further split experiments as a test dose. The treatment was performed for 1 min on ice during centrifugation (825× *g*), as previously described by us [15,16,17].

Step 2: Effect of the intertreatment time (ITT) on cell survival and DSB levels:

Cells were pretreated with 10 μg/mL, washed with YEPD, and different ITTs—0, 30, 45, 60, or 120 min ITT—were given at an optimal for cell culture conditions (30 °C with aeration at 200 rpm) and on ice (to suppress activation of defense mechanisms, including DSB repair processes). After that, the cell suspensions were treated with the test dose of Zeo (100 μg/mL) and subsequently subjected to microbiological and molecular analyses to determine cell survival, DNA double-strand breaks in both strains, mitotic gene conversion, reverse mutations, and total aberrant events in D7ts1. As a result of these experiments, the optimal ITT time (45 min) between priming and the test dose was determined.

**Figure 6:**
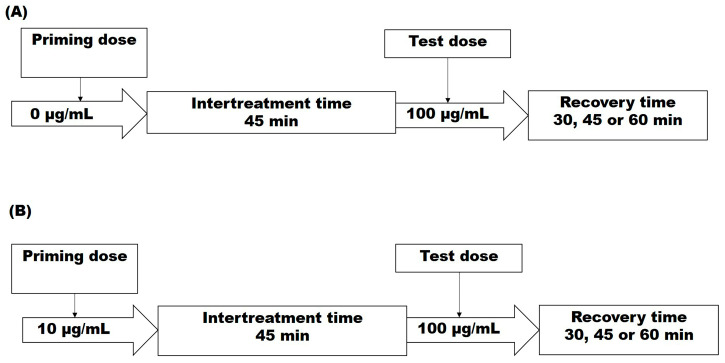
Experimental schemes for the evaluation of the contribution of recovery time (RT) after: (**A**) single test dose treatment; (**B**) experimental design for formation of an adaptive response measured as residual DNA damage (lesions) represented by double-strand breaks (DSBs).

Step 3: The contribution of the recovery time to the DSB levels in both strains D7ts1 and 551:

Based on the results obtained in steps 1 and 2, the schemes shown below were used (Figure 6):

In short, cells were pretreated with the priming dose as described above, washed with YEPD, and then subjected to a 45 min ITT under optimal growth conditions (30 °C, aeration at 200 rpm). After that, the samples were treated with the test dose of Zeo. After washing the cells with YEPD, they were incubated for 30, 45, and 60 min (recovery time), and the DSB repair capacity was measured as a fraction of damage remaining (FDR).

### 4.5. Microbiological Method

#### Cell Survival

In survival experiments, cells were plated immediately after the test dose treatment following the scheme: priming dose >>> ITT >>> test dose >>> plating. Briefly, after the treatments described as steps 1 and 2, cell suspensions were washed. Appropriate dilutions of cell suspensions for both strains were plated onto a solid YEPD medium (1% yeast extract, 2% peptone, 2% glucose, and 2% agar) to assess survival [16]. Cell survival was counted after 3 days. Control untreated cells were taken as 100% cell survival.

Zimmermann’s test [82] with diploid strain D7ts1 (*MATa/α ade2-119/ade2-40 trp5-27/trp5-12 ilv1-92/ilv1-92 ts1/ts1*) was applied. The D7ts1 strain provides simultaneous detection of mitotic gene conversion at the *trp-5* locus, reversion mutations at the *ilv1* locus, and mitotic crossing-over between the centromere and the *ade2* allele [84]. All genetic events linked with the *ADE2* locus are classified as total aberrations [90,91].

Briefly, after reaching the appropriate growth phase, cells were harvested and resuspended in 1× PBS (Phosphate-Buffered Saline). The cell suspension was then treated with 10 µg/mL Zeo for 1 min on ice during centrifugation (825× *g*). Cells were resuspended and incubated for 45, 60, or 120 min under optimal growth conditions (30 °C, 200 rpm) or on ice (to inhibit repair). Subsequent treatment with 100 µg/mL for 1 min on ice during centrifugation was performed. The pellet was resuspended in 1× PBS. Appropriate dilutions of cell suspensions were plated onto a solid complete medium to assess survival and total aberrants. Gene conversion was detected on selective media lacking tryptophan, and selective media lacking isoleucine were used for reverse mutations. Five plates in each category were incubated for 5–7 days at t = 30 °C. Yeast media were prepared as described by Zimmermann et al. [82].

### 4.6. Molecular Method

The DSBs’ rejoining capacity was assessed by constant-field gel electrophoresis (CFGE) using the optimized *Saccharomyces cerevisiae* protocol [16,17].

Briefly, after the treatments described above, cells were washed twice, and the pellet was inserted into the agarose plugs. Subsequently, cell lysis was performed using a lysis solution containing 1 mg/mL Proteinase K for 20 h. Electrophoresis was performed in 0.5× TBE buffer (Tris-Borate-EDTA) for 40 h at a constant field strength of 0.6 V/cm. The levels of induced DSBs, presented as a fraction of DNA released (FDR) from the wells, were quantified by measuring ethidium bromide fluorescence using the Gene Tool Analyzer G: Box Syngene (software version 4.3.17.0).

### 4.7. Data Analysis

The experiments were repeated at least three times from independently grown cultures. Data points in all the figures are mean values. Error bars represent standard errors of mean values. Where no error bars are evident, errors were equal to or less than the symbols. Surviving Fraction (SF) was calculated as follows: SF = % survival colonies in treated samples/% survival colonies in untreated samples. Normalized split dose (NSD) was calculated as NSD = SF A + D/SF D, where A is the adaptive/priming dose, and D is the damaging (test dose) [92]. The fraction of DNA damage released (FDR) was calculated as FDR = DNA released/DNA in the wells + DNA released [93]. The normalized FDR = FDR after AR treatment/FDR after single test dose treatment [14,25,26]. The magnitude of the adaptive response was calculated as the Area Under the Curve (ΔAUC). It represents the difference between the single treatment with the test dose (AUC_D_) and after split treatment with both doses (AUC_A+D_): ΔAUC = AUC_D_ − AUC_A+D_ [94]. All calculations were performed using GraphPad Prism, version 9.5.1 (San Diego, CA, USA). The statistical analyses were performed using one-way and two-way ANOVA with Tukey’s post hoc test. Linear correlation was assessed using the Pearson Product-Moment Correlation Coefficient (r). Asterisks provide information about the significance of the differences, where * *p* < 0.05; ** *p* < 0.01; *** *p* < 0.001; and **** *p* < 0.0001.

## 5. Conclusions

In this work, favorable experimental conditions for AR induction in both D7ts1 and 551 strains were described: the priming dose, which could induce about 20% lethality or at least a 1.5-fold increase in DSB levels, the inter-treatment time interval (ITT)—45 min, and the recovery time—30–45 min. Both strains developed well-expressed AR, measured as an increased cell survival, but differed in their ability to develop AR, measured as a reduction in DSBs. The main reason is probably not due to their DNA susceptibility, but rather to differences in their capacity to repair DSBs.

The influence of this design on the frequency of total aberrants, mitotic gene conversion, reverse mutations, and DSB rejoining provides indirect evidence for the involvement of various DNA repair mechanisms and for chromatin reorganization in the formation of an adaptive response.

The incomplete correspondence between results obtained using two different endpoints (SF and acceleration of DSB rejoining) to assess the adaptive response prompted us to make two main assumptions: DSB repair is probably only partially responsible for the higher cellular survival in the split experiments, and secondly, it would be good to examine the adaptive response according to at least two criteria.

Our findings show that the development of an AR depends on several factors: the magnitude of the priming dose, DNA susceptibility, the duration of the ITT window, the duration of recovery time, as well as genetic constitution of strains.

## Figures and Tables

**Table 1:** Magnitude of adaptive response in strain D7ts1, depending on the recovery time, given as 30, 45, and 60 min after the test dose.

RT (min)	ΔAUC ^1^
30	1.027
45	1.419
60	1.068

^1^ ΔAUC is calculated with GraphPad Prism Software and represents the difference between the areas under curves of single treatment with the test dose (AUC_D_) and after split treatment with both doses (AUC_A+D_) in Figure 5.

**Table 2:** The frequency of mitotic gene conversion at the *trp5* locus, reversion of the *ilv1–92* allele, and total aberrants in the *ade2* locus after single and split treatment of strain D7ts1 with Zeo.

Samples ^1^			Convertants/10^5^ Cells	Revertants/10^6^ Cells	Total Aberrants (%)
Single treatments	Control		0.41 ± 0.04	0.011 ± 0.001	0.08 ± 0.001
	Priming dose (10 μg/mL)		1.35 ± 0.02 ***	0.028 ± 0.005 *	0.20 ± 0.014 ***
	Test dose (100 μg/mL)		12.75 ± 0.34 ****	0.092 ± 0.005 ****	2.88 ± 0.157 ****
Split treatments	Optimal temperature	45 min ITT	6.65 ± 0.02 ****	0.036 ± 0.003 ***	0.43 ± 0.025 ****
	On ice	45 min ITT	7.83 ± 0.01 ****	0.066 ± 0.003 ****	0.82 ± 0.030 ****

^1^ The statistical significance of the differences for each endpoint was assessed using a one-way ANOVA with Tukey’s post hoc test (* *p* < 0.05; *** *p* < 0.001; **** *p* < 0.0001). Nine independent experiments were performed. The results were evaluated on day 6 after the Petri dishes were plated.

**Table 3:** Correlation analysis to evaluate the potential relationship among the investigated markers when a split Zeo treatment at optimal temperature was performed.

Studied Markers ^1^	Cell Survival	Mitotic Gene Conversion	Reverse Mutations	Total Aberrants	DSBs
Cell survival	-	−0.898	−0.899	−0.875	−0.957 *
Mitotic gene conversion		-	0.944	0.991 **	0.987 **
Reverse mutations			-	0.970 *	0.947 *
Total aberrants				-	0.971 *
DSBs					-

^1^ Data are presented as a correlation coefficient (r). Linear correlation was assessed using the Pearson Product-Moment Correlation Coefficient (r). An “r” higher than −0.900 denotes a strong correlation. Asterisks indicate the statistical significance of the correlation coefficient (* *p* < 0.05; ** *p* < 0.01).

**Table 4:** Correlation analysis to evaluate the potential relationships among the investigated markers when a split Zeo treatment was performed on ice.

Studied Markers ^1^	Cell Survival	Mitotic Gene Conversion	Reverse Mutations	Total Aberrants	DSBs
Cell survival	-	−0.847	−0.885	−0.868	−0.960 *
Mitotic gene conversion		-	0.991 **	0.939	0.929
Reverse mutations			-	0.909	0.967 *
Total aberrants				-	0.861
DSBs					-

^1^ Data are presented as a correlation coefficient (r). Linear correlation was assessed using the Pearson Product-Moment Correlation Coefficient (r). An “r” higher than −0.900 denotes a strong correlation. Asterisks indicate the statistical significance of the correlation coefficient (* *p* < 0.05; ** *p* < 0.01).

## Data Availability

The original contributions presented in this study are included in the article. Further inquiries can be directed to the corresponding author.

## Abbreviations

The following abbreviations are used in this manuscript:

Zeo	Zeocin^TM^
DDR	DNA damage response
AR	Adaptive response
DSB	Double-strand break
SF	Survival fraction
ITT	Intertreatment time
NSF	Normalized survival fraction
FDR	Fraction of damage released
RT	Recovery time
AUC	Area under curve
